# Increased growth temperature and vitamin B12 supplementation reduces the lag time for rapid pathogen identification in BHI agar and blood cultures

**DOI:** 10.12688/f1000research.129668.1

**Published:** 2023-02-03

**Authors:** Jawad Ali, Mukund Joshi, Asal Ahmadi, Knut Olav Strætkvern, Rafi Ahmad

**Affiliations:** 1 Department of Biotechnology, Inland Norway University of Applied Sciences, Hamar, Norway; 2 Institute of Clinical Medicine, UiT - The Arctic University of Norway, Tromsø, Norway

**Keywords:** Lag time, Growth temperature, Vitamin B12, Brain heart infusion, Antimicrobial resistance, Pathogen detection, Blood culture

## Abstract

**Background:** The rapid diagnostics of pathogens is essential to prescribe appropriate and early antibiotic therapy. The current methods for pathogen detection require the bacteria to grow in a culture medium, which is time-consuming. This increases the mortality rate and the global burden of antimicrobial resistance. Culture-free detection methods are still under development and are not used in the clinical routine. Therefore decreasing the culture time for accurate detection of infection and resistance is vital for diagnosis.

**Methods:** In this study, we wanted to investigate easy-to-implement factors (in a minimal laboratory set-up), including inoculum size, incubation temperature, and additional supplementation ( *e.g.*, vitamin B12 and trace metals), that can significantly reduce the lag time (t _lag_). These factors were arranged in simple two-level factorial designs using Gram-positive ( *Escherichia coli* and *Pseudomonas aeruginosa*) and Gram-negative ( *Staphylococcus aureus* and *Bacillus subtilis*) bacteria, including clinical isolates with known antimicrobial resistance profiles. Blood samples spiked with a clinical isolate of *E. coli* CCUG17620 were also tested to see the effect of elevated incubation temperature on bacterial growth in blood cultures.

**Results: **We observed that increased incubation temperature (42°C) along with vitamin B12 supplementation significantly reduced the t _lag_ (10 – 115 minutes or 4% - 49%) in pure clinical isolates and blood samples spiked with *E. coli* CCUG17620. In the case of the blood sample, PCR results also detected bacterial DNA after only 3h of incubation and at three times the CFU/mL.

**Conclusions:** Enrichment of bacterial culture media with growth supplements such as vitamin B12 and increased incubation temperature can be a cheap and rapid method for the early detection of pathogens. This is a proof-of-concept study restricted to a few bacterial strains and growth conditions. In the future, the effect of other growth conditions and difficult-to-culture bacteria should be explored to shorten the lag phase.

## Introduction

The discovery of antibiotics was a breakthroughs in therapeutic medicines, which has enabled us to treat critical bacterial infections ( Ali *et al.*, 2020). But even before the introduction of the first antibiotic into clinical practice, some bacteria had already become resistant ( D’Costa *et al.*, 2006). The ability of bacteria to survive in the presence of antibiotics which were earlier used to treat the infections caused by these bacteria, is called “antimicrobial resistance (AMR)” ( Kapoor *et al.*, 2017). The misuse of antibiotics in clinical, agriculture, poultry, and aquaculture sectors is significantly increasing the emergence of AMR. The World Health Organization (WHO) has described AMR as a global health issue. Infections due to antibiotic resistant bacteria are rising. In 2019, 1.27M and 4.95M deaths were directly caused or associated with antibiotic-resistant bacteria, respectively ( Murray *et al.*, 2022). It is predicted that at the current rate, AMR will cause 10 million deaths per year and cost $100 trillion to the world economy.

The discovery void of new antibiotics also adds to the growing AMR problem. It has been more than 35 years since a new class of antibiotics was discovered, and bacteria are resistant to almost all the presently used antibiotics ( Hoffman, 2020). The unavailability of new antibiotics further increases the need for rapid diagnostics of pathogens and their AMR. Early and appropriate antibiotic therapy is crucial in cases of severe bacterial infections and can significantly reduce mortality. Sepsis is considered a major health problem and accounts for an estimated 31.5 million cases with a mortality rate of 5.3 million deaths globally per year ( Fleischmann *et al.*, 2016; Taxt *et al.*, 2020). In the case of sepsis, early treatment with appropriate antibiotics is highly vital for the patient’s survival ( Bloos *et al.*, 2017; Ferrer *et al.*, 2014). In septic patients, delaying each hour the prescription of appropriate antibiotics can have lethal consequences ( Avershina *et al.*, 2022). Due to the lack of adequate and early diagnostics, clinicians are pushed to prescribe empirical antibiotics to the patients, which in most cases are not very effective and further disseminate antibiotic resistance ( Khaledi *et al.*, 2020).

To decrease the use of empirical and broad-spectrum antibiotics, optimizing the existing diagnostic methods and making them faster and more cost-effective is crucial. The current diagnostic procedures still rely on culture-based techniques where bacteria are cultured and then identified using different biochemical tests. After that, antibiotic susceptibility testing (AST) is performed, which gives an idea about the resistance or sensitivity of bacteria to different antibiotics. The culture-based methods have a turnaround time of two to four days in most samples. However, in the case of bloodstream infections where the bacterial load is lower, the identification and AST can take up to five days ( Briggs *et al.*, 2021; Metzgar *et al.*, 2016). There are many emerging micro- and nanotechnologies for pathogen identification and AST, including both phenotypic (microfluidic-based bacterial culture) and molecular methods (PCR, hybridization probes, nanoparticles, synthetic biology, and mass spectrometry) ( Li *et al.*, 2017). Whole genome sequencing (WGS) using the Oxford Nanopore Technology’s (ONT) MinION has the potential to detect pathogens and their associated antibiotic resistance genes (ARGs) rapidly, but this technique is still in development, is costly, and not used in clinical settings ( Avershina *et al.*, 2022; Taxt *et al.*, 2020). All these techniques are expensive, need dedicated infrastructure, or are time-consuming. Therefore, there is an urgent need to develop cost- and resource-effective methods that can speed up pathogen diagnostics and reduce the mortality from these infections.

Mesophilic bacteria are typically grown at 30 to 37°C in lab procedures. In rich growth media ( *i.e.*, Luria-Bertani, Terrific Broth), dense cultures are raised in a reasonably short time of two to six hours using inoculums 1-2% of overnight cultures (ca. 12-14 h). When growing bacteria from clinical samples in blood cultures, however, a far lower inoculum is experienced, *i.e.*, <100 CFU or 0.5 McFarland units (MFU), and growth must be pursued for a minimum of 24 h up to several days to detect potential pathogens ( Doualeh *et al.*, 2022; Hardy *et al.*, 1992; Waldeisen *et al.*, 2011). The lengthy growth of fastidious bacteria in blood cultures to reach detectable titers is a significant bottleneck in the workflow of testing pathogens for AMR ( Menchinelli *et al.*, 2019; Somily *et al.*, 2018; Verroken *et al.*, 2015). Brain heart infusion (BHI) is a nutrient growth medium suitable for blood culture and to culture various fastidious organisms. BHI broth is often used in several application areas, including clinical microbiology testing, life science, biopharmaceuticals, food safety, water safety, and antibiotic sensitivity tests.

The bacterial growth curve shows four phases: lag phase, log (exponential) phase, stationary, and death phase. The lag phase is also called the preparatory phase, where the bacteria adapt to the new environment, and the cells are metabolically active. The log phase is where the bacterial cells double and increase in number until the depletion of nutrients. The log phase is followed by the stationary phase, where the bacterial growth rate equals the death rate due to the accumulation of toxic compounds and nutrient depletion. The final phase is called the death phase, in which the death rate of bacteria is greater than the growth rate.

Clearly, the time for a culture to reach detectable density, *i.e.*, the length of lag phase growth, is a bottleneck in the diagnostic workflow. In this study, we wanted to investigate easy-to-implement factors (in a minimal laboratory set-up), including inoculum size, incubation temperature, and additional supplementation ( *e.g.*, vitamin B12 and trace metals), that can significantly reduce the lag time (t _lag_). These factors were arranged in simple two-level factorial designs using different Gram-positive ( *Escherichia coli* and *Pseudomonas aeruginosa*) and Gram-negative ( *Staphylococcus aureus* and *Bacillus subtilis*) bacteria, including clinical isolates with known AMR profiles (encoding methicillin resistance or extended-spectrum β-lactamases). BHI was used as the growth medium as it is suitable for use as a blood culture medium. Based on the results from experiments with pure bacterial isolates, blood samples spiked with a clinical isolate of *E. coli* CCUG17620 were also tested to see the effect of elevated incubation temperature on bacterial growth in blood cultures.

## Methods

### Bacterial strains

The study focused on seven different bacterial strains covering four different bacterial species. Clinical bacterial strains *E. coli* NCTC13441 and *S. aureus* NCTC8325 were obtained from the National Collection of Type Cultures (NCTC), while *S. aureus* CCUG35600 and *E. coli* CCUG17620 were obtained from the Culture Collection University of Gothenburg (CCUG). Three non-clinical wild-type strains, including *E. coli* HB101K2 *, Pseudomonas aeruginosa INN*, and *Bacillus subtilis INN,* were taken from the internal bacterial collection of Inland Norway University (INN). Additional details are available in Table 1.

**Table 1.** An overview of the different combinations of experiments performed, including information regarding different bacterial strains, their antibiotic susceptibility phenotype, and growth variables ( *i.e.*, Temperature, Inoculum size, and vitamin B12 supplementation) symbolised X _1_, X _2_, and X _3_.

Experiment No.	Bacterial strain	Antibiotic susceptibility phenotype	Gram staining	Temperature (°C) X _1_	Inoculum size (v/v %) X _2_	Vitamin B12 (μM) X _3_
1	*Escherichia coli* HB101 K2	wild type	negative	37	0.05	0
2				37	0.05	50
3				37	0.5	0
4				37	0.5	50
5				42	0.05	0
6				42	0.05	50
7				42	0.5	0
8				42	0.5	50
9	*Staphylococcus aureus* NCTC8325 ^*^	wild type	positive	37	0.05	0
10				37	0.05	50
11				37	0.5	0
12				37	0.5	50
13				42	0.05	0
14				42	0.05	50
15				42	0.5	0
16				42	0.5	50
17	*Pseudomonas aeruginosa* INN	wild type	negative	37	0.5	0
18				37	0.5	50
19				42	0.5	0
20				42	0.5	50
21	*Bacillus subtilis* INN	wild type	positive	37	0.5	0
22				37	0.5	50
23				42	0.5	0
24				42	0.5	50
25	*E. coli* CCUG17620 ^*^	wild type	negative	37	0.5	0
27				42	0.5	0
29	*E. coli* NCTC13441 ^*^	resistant (ampicillin, extended-spectrum cephalosporins, ciprofloxacin, trimethoprim)	negative	37	0.5	0
31				42	0.5	0
33	*S. aureus* CCUG35600 ^*^	resistant (methicillin, tetracycline, clindamycin, erythromycin)	positive	42	0.5	0
35				42	0.5	50

^*^ Clinical strains.

### Chemicals

All reagents were obtained from standard lab suppliers. Bacteriological agar and Brain Heart Infusion (BHI) broth for the media preparation were the products of HiMedia Laboratories (Mumbai, India; lot number: BCBH4004V), with BHI consisting of (g/L) calf brain infusion from 200 g (12.5), beef heart infusion from 250 g (5), peptone (10), sodium chloride (5), disodium chloride D (+) glucose (2), disodium hydrogen phosphate (2.5) at pH 7.4. Vitamin B12 or cyanocobalamin was from ThermoFisher, ( Massachusetts, USA; number A14894). In addition, we also tested three clinical isolates using a trace metal solution (details in Supplementary Table 1, *Extended data* [ Ahmad 2023]).

### Pre-culture and growth procedures

BHI agar and liquid culture media were used to maintain cultures and to examine growth curves. Bacterial samples stored at -80°C were thawed gently at room temperature, inoculated to BHI broth, and incubated overnight at 37°C. BHI agar plates were prepared by adding 1.5% bacteriological agar powder to BHI broth. An overnight inoculum was streaked out, and plates were incubated at 37°C for 24 h. Single colonies were selected and inoculated into fresh BHI broth. The inoculated BHI broth was incubated at 37°C overnight and served as inoculum for culture experiments (See Supplementary figure S1 for an overview of the complete process, *Extended data*). Experimental liquid cultures were cultivated in shake flasks (500 mL broth in 2L Erlenmeyer bottles). Cultures within the same experimental design were inoculated from the same bacterial preculture suspension to ensure identical starting conditions.

All cultures in BHI broth were incubated at 37°C or 42°C under shaking conditions for up to 6 h, whereas blood cultures were cultivated for 12 h. During incubation, the optical density (OD) was measured at 600 nm every 20-30 minutes, starting from the time of inoculation (0 min) until the bacterial growth reached the stationary phase (approximately 4-5 h). Culture aliquots were immediately cooled and kept on ice until measurement. The OD 600 was plotted *versus* time (min) to obtain the growth curves.

**Figure 1.**
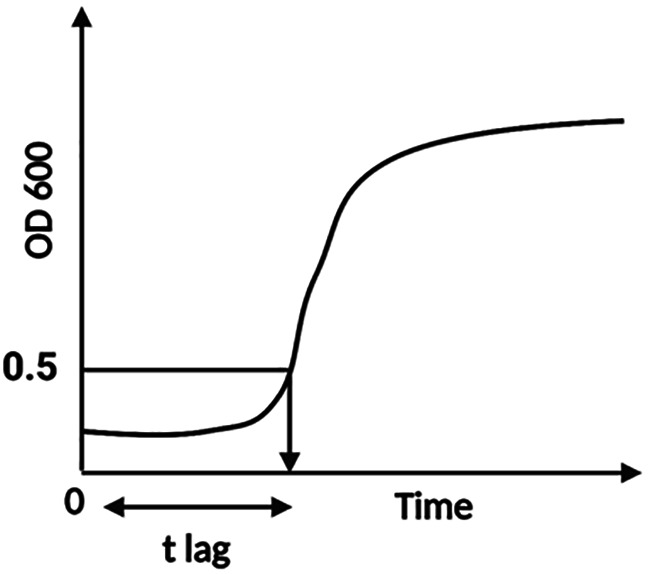
Graphic description of the determination of t _lag_ from the growth curves used in this study. The 0.5-intercept represents the net increase in OD 600, *i.e.*, the value subtracted from the optical density (OD) at time zero.

As a practical response value to estimate the effectiveness of treatments, an endpoint of the lag time (t _lag_) was defined, the t _lag_ being the time (min) from inoculation until the OD 600 value reached a net increase of 0.5. The net OD increase was obtained by subtracting the OD at t=0 from all OD values. The t _lag_ was obtained by extrapolating at OD 0.5 from the zero-adjusted growth curve. Although this provisional end point is beyond the actual end of lag ( Bertrand, 2019), an extended OD cut-off value was selected to reduce the estimation error when growth curves go from an undefined shallow rise to a steady exponential growth ( Figure 1).

### Design of experiments for screening of growth variables

A set of growth screening experiments examined the ability of selected variables to cause a reduced lag time. A structured two-level factorial design (Design of Experiments, DOE) was used to observe the effect of incubation temperatures (37°C and 42°C), low (0.05%), and high (0.5%) inoculum, vitamin B12 (±50 μM), and trace metal (present y/n) supplements. The design was tested on the wild-type strains (3) and the clinical isolates (4) but with a selection of two or three variables or factors. Table 2 shows the coded and decoded values of the factors tested (X _1_, X _2_, X _3_) at a low (-1) and high (+1) level. Thus, either four (2 ^2^) or eight (2 ^3^) growth experiments were performed for each strain. For a detailed overview of conditions for the individual strains, Table 1 is provided as well as in Supplementary Tables 2-4 ( *Extended data*).

**Table 2.** The coded and uncoded variables for screening the growth behavior in bacterial cultures.

Variable	Factor	Levels	
		-1	+1
Temperature (°C)	X _1_	37	42
Inoculum size (v/v %)	X _2_	0.05	0.5
Vitamin B12 (μM)	X _3_	0	50

The estimated t _lag_ values from DOE were presented in interaction effect plots, illustrating the impact of a variable ( *e.g.*, temperature) with connected experiments on another variable ( *e.g.*, vitamin B12 supplement). The main effects of tested variables are shown numerically in descending order in bar charts. The bars are the absolute effect values computed as twice the regression coefficients from fitted DOE models. They show the change in t _lag_ when a variable goes from a low to a high level when other factors are kept at their average.

### Blood culturing

Clinical strain *E. coli* CCUG17620 was spiked with blood to mimic the sepsis infection. The blood was spiked with 40 CFU/mL from an overnight plate culture of the clinical strain. The lower CFU was used as an inoculum because, in the case of sepsis, the CFU/mL ranges typically from 10-100 ( Doualeh *et al.*, 2022). *E. coli* was grown overnight on a BHI agar plate and then suspended in saline to measure the OD. After measuring OD, the suspensions were plated on agar plates to calculate the CFU at different OD values. After optimizing the relation of OD with CFU counts, the desired concentration in the range of 10-100 CFU/mL was used for spiking with human blood. Fresh human blood obtained from healthy donors at Inland Norway University was used for this experiment. Human blood spiked with *E. coli* was mixed with BHI broth medium and incubated at the two study temperatures, *i.e.*, 37 and 42°C. CFU/mL was calculated every 3 h for 12 h by plating out the samples on BHI agar plates.

### DNA extraction and PCR from blood cultures

Bacterial DNA from blood culture samples was extracted using QIAamp BiOstic Bacteremia DNA Kit from Qiagen (Germany). The concentration and purity of isolated DNA were determined using the Qubit system with a dsDNA HS assay kit (Thermo Fisher Scientific, USA) and the NanoDrop spectrophotometer. PCR was performed to confirm the presence of *E. coli* CCUG17620 DNA and human DNA in samples taken at 3 h, 6 h, 9 h, and 12 h of incubation at 37°C and 42°C. The primers used for identifying *E. coli* were uspA-F CCGATACGCTGCCAATCAGT and uspA-R ACGCAGACCGTAGGCCAGAT, and human DNA was β-actin (F-CGGCCTTGGAGTGTGTATTAAGTA and R-TGCAAAGAACACGGCTAAGTGT) ( Hasan *et al.*, 2016). The PCR reaction was set up in a total volume of 20 μL per sample containing 4 μL HOT FIREPol MultiPlex Mix Ready to Load master mix from Solis Biodyne (Estonia), 0.5 μL of 10 μM forward and reverse primers, 8 ng/μL of template DNA and nuclease-free water. The thermal cycling conditions for PCR were initial denaturation at 95°C for 12 min followed by 35 cycles of 95°C for 25 s, 61°C for 50 s and 72°C for 1 min, and a final extension of 72°C for 7 min. The PCR product was run on 1% agarose gel for 45 min at 100V and then visualized on the Gel doc system to determine the presence or absence of our desired bands.

### Statistical analysis

The MODDE 13.0 Pro (Umetrics/Sartorius Stedim Data Analytics AB, Sweden) was used to generate the design matrix, regression coefficients, and statistical analysis. The significance testing (p < 0.05) of the DOE screening was performed with ANOVA. All the growth curve graphs are generated using GraphPad Prism 9.4.1.

## Results

### Higher incubation temperature, along with vitamin B12 supplementation, reduces the lag time for bacterial growth

*Escherichia coli*

*E. coli* HB101 K2

**Figure 2.**
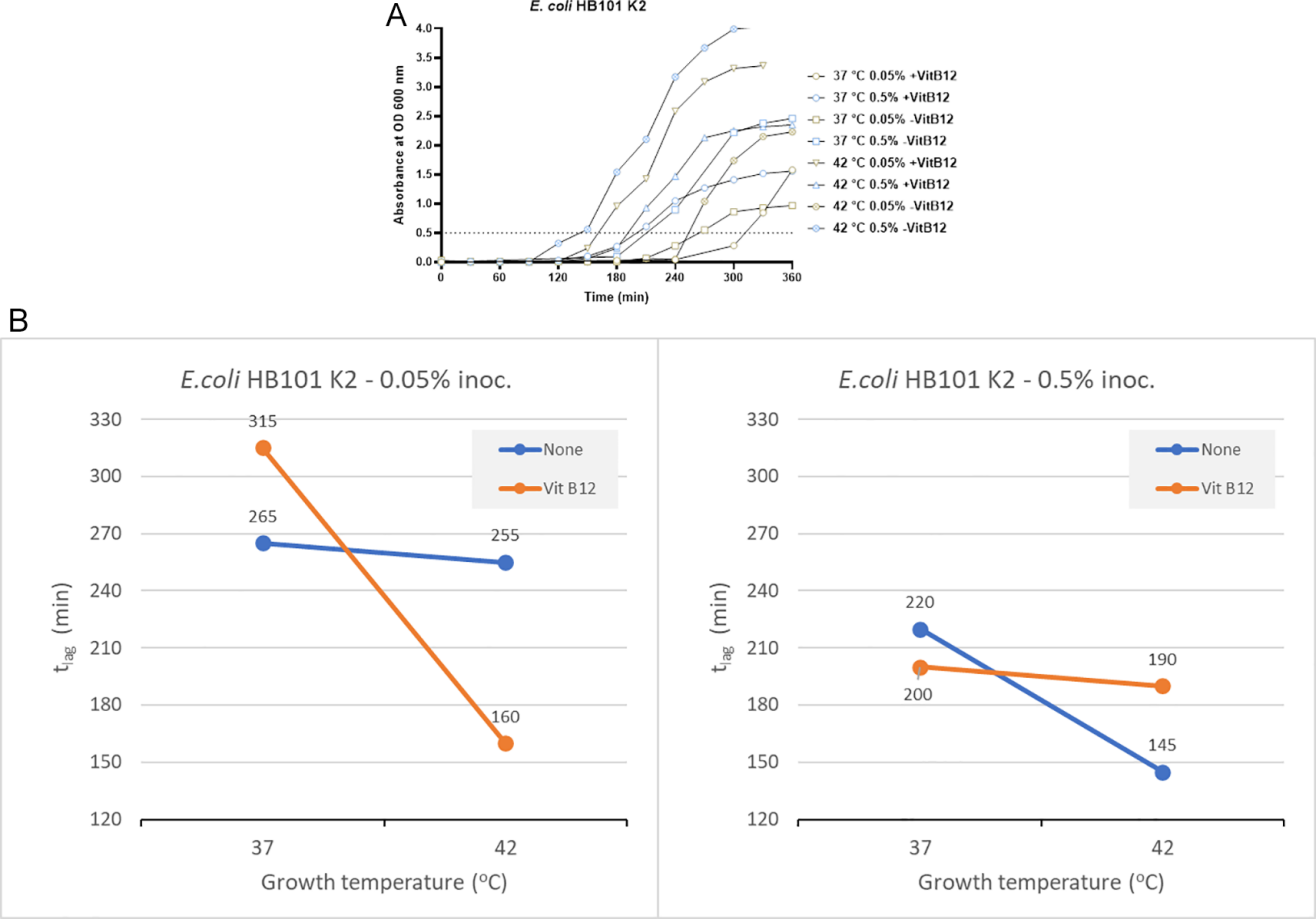
Results from *E. coli* HB101K2 under different growth conditions, including inoculum size, temperature, and vitamin B12 supplementation. (A) Growth curves of *E. coli* HB101K2 were obtained in three-factorial designs; at 37°C and 42°C, inoculum size 0.05% and 0.5 %, with and without vitamin B12. The dotted line indicates OD 0.5 for estimating the t _lag_ from curves. (B) Effect plot of *E. coli* HB101 K2 in minutes (t _lag_) to reach optical density 0.5.

In the absence of vitamin B12 supplementation, the t _lag_ for bacterial growth was 265 minutes and 255 minutes at 37°C and 42°C, respectively, when an inoculum size of 0.05% was used for culturing ( Figure 2A). When using a higher inoculum of 0.5% in the absence of vitamin B12 supplement, the t _lag_ recorded was 220 minutes and 145 minutes at 37°C and 42°C, respectively ( Figure 2A). In the presence of vitamin B12 supplement and using an inoculum size of 0.05%, the bacterial t _lag_ was 315 minutes (37°C) and 160 minutes (42°C). Similarly, when the inoculum size was increased to 0.5%, with vitamin B12 supplementation, the t _lag_ was 200 minutes (37°C) and 190 minutes (42°C). The shortest t _lag_ was recorded to be 145 minutes, which was observed using an inoculum size of 0.5% in the absence of vitamin B12 supplement and incubation at 42°C ( Figure 2 and Supplementary Table 2 [ *Extended data*]).

Using an inoculum size of 0.05% and a vitamin B12 supplement, we observed 95 minutes of reduction in the lag time at 42°C compared to 37°C ( Figure 2B). In contrast, when the inoculum size was increased to 0.5% along with vitamin B12 supplementation, the t _lag_ was reduced by 10 minutes at 42°C. Based on the effect plot, it is reported that the sample with no vitamin B12 supplement and 0.5% inoculum size showed the shortest t _lag_. But when the inoculum size was decreased to 0.05%, we observed that the sample incubated at 42°C in the presence of vitamin B12 supplement showed the shortest t _lag_ ( Figure 2B). The reduction in t _lag_ was between 4% and 49% for the above mentioned eight tested conditions.

*E. coli* CCUG17620 and *E. coli* NCTC13441

**Figure 3.**
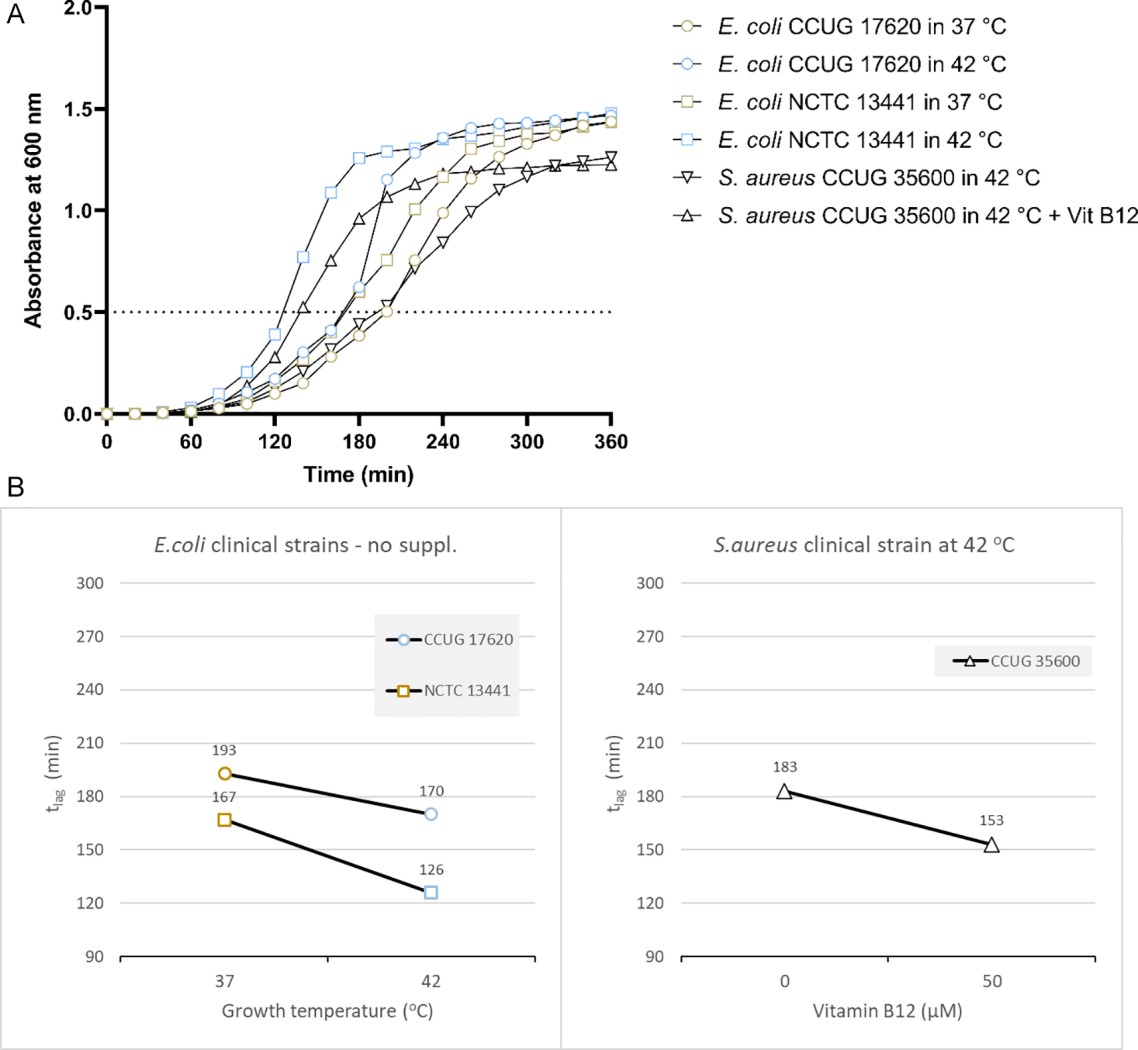
Results for the clinical strains *E. coli* CCUG17620 *, E. coli* NCTC13441, and *S. aureus* CCUG35600 under different growth conditions, including temperature, and vitamin B12 supplementation. (A) Growth curves of clinical bacterial isolates at 37 °C and 42 °C ( *E. coli* CCUG17620, *E. coli* NCTC13441), or with and without vitamin B12 supplement ( *S. aureus* CCUG35600 at 42 °C). The dotted line indicates OD 0.5 for estimating the t _lag_ from curves. (B) Effect plot of *clinical samples* in minutes (t _lag_) to reach optical density 0.5.

Based on the results from the laboratory strain, we tested the growth of two *E. coli* clinical isolates at 37 and 42°C. *E. coli* CCUG17620 took 170 minutes when incubated at 42°C and 193 minutes at 37°C to reach OD 0.5 without adding vitamin B12. Similarly, *E. coli* NCTC13441 required 126 and 167 minutes to reach OD 0.5 at 42°C and 37°C, respectively ( Figure 3A and Supplementary Table 4 *[Extended data*]). For the two clinical isolates, *E. coli* CCUG17620 and *E. coli* NCTC13441, the t _lag_ was less at 42°C compared to 37°C.

Overall, the effect plot of *E. coli* CCUG17620 *and E. coli* NCTC13441 at inoculum size 0.5% indicated that when the incubation temperature increased (42°C instead of 37°C), the lag time decreased by 15-25% in the tested strains ( Figure 3B).

*Staphylococcus aureus*

*S. aureus* NCTC8325

**Figure 4.**
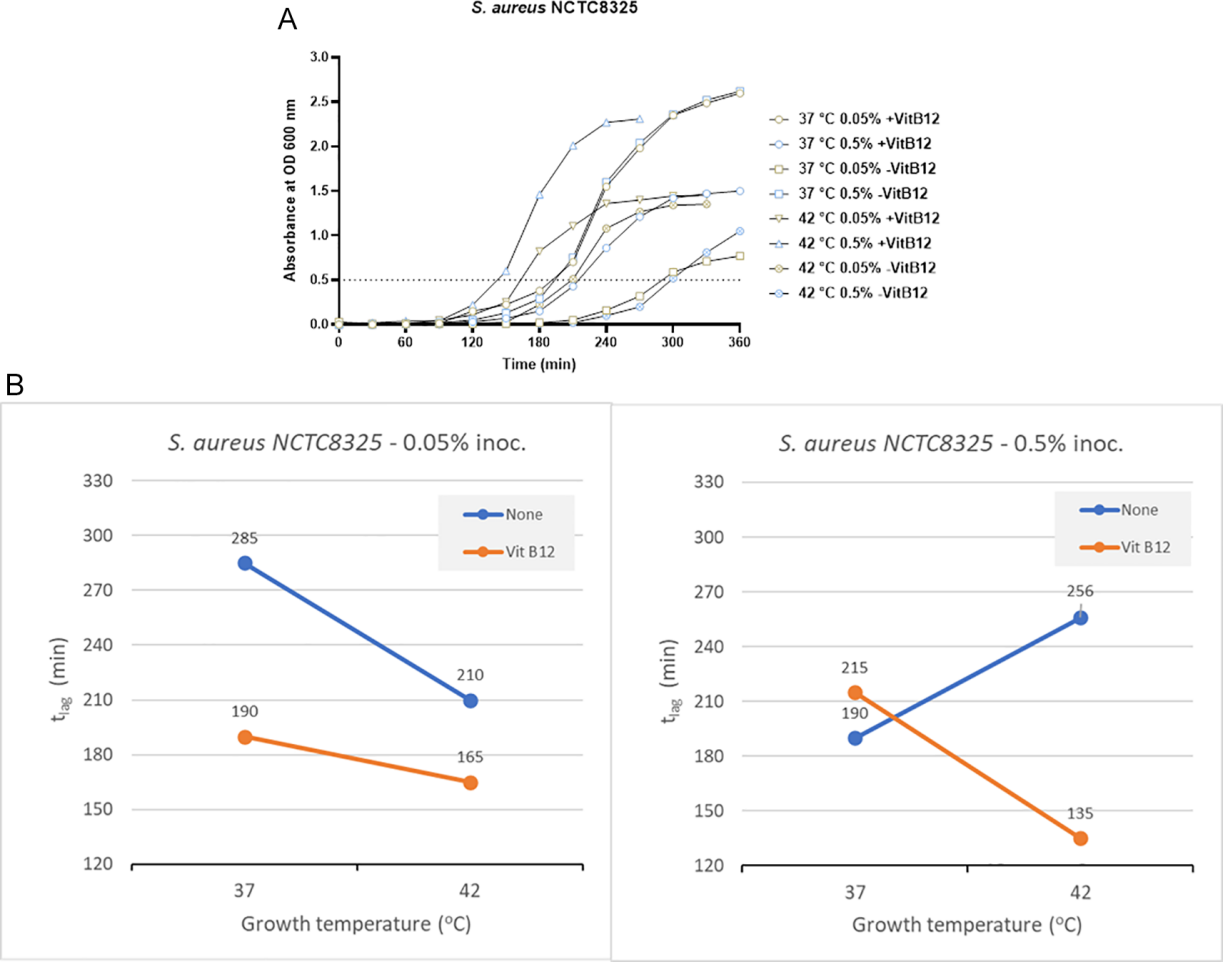
Results for *S. aureus* NCTC8325 under different growth conditions, including inoculum size, temperature, and vitamin B12 supplementation. (A) Growth curves of *S. aureus* NCTC8325 were obtained in three-factorial designs; at 37°C and 42°C inoculum sizes 0.05% and 0.5 %, with and without vitamin B12. The dotted line indicates OD 0.5 for estimating the t _lag_ from curves. (B) Effect plot of *S. aureus* NCTC8325 in minutes (t _lag_) to reach optical density 0.5.

In the case of the clinical isolate *S. aureus* NCTC8325 with no vitamin B12 supplements and 0.05% inoculum size, the time to reach OD 0.5 was 285 minutes and 210 minutes at 37°C and 42°C, respectively ( Figure 4A and Supplementary Table 2). But in the presence of vitamin B12 supplementation and lower inoculum size of 0.05%, the t _lag_ was 190 minutes (37°C) and 165 minutes (42°C). When the inoculum size was increased to 0.05%, the t _lag_ decreased significantly at 42°C (135 minutes) in comparison to 37°C (215 minutes) in the presence of vitamin B12 supplementation ( Figure 4A and Supplementary Table 2). We can conclude that incubation at 42°C, along with vitamin B12 supplement, reduces the t _lag_ in *S. aureus* NCTC8325.

The effect plot of *S. aureus* NCTC8325 at 0.5% inoculum shows a reduction of 121 minutes in t _lag_ at 42°C incubation in the presence of vitamin B12 supplement. At the same time, a reduction of 45 minutes in t _lag_ was observed at 0.05% inoculum and 42°C incubation in the presence of vitamin B12 supplement ( Figure 4B). The reduction in t _lag_ was between 13% and 37% for the eight tested conditions.

*S. aureus* CCUG35600

Based on the above results, we tested the growth of another clinical strain of *S. aureus.* The effect plot of the clinical isolate *S. aureus* CCUG35600 at inoculum size 0.5% at 42°C co-cultured with vitamin B12 supplement showed a decline in lag time compared to without supplementation. Overall, the shortest t _lag_ time was observed following the co-culture of bacteria with vitamin B12 supplement ( Figure 3A and B). So, the reduction in t _lag_ was 20%.

*Pseudomonas aeruginosa*

**Figure 5.**
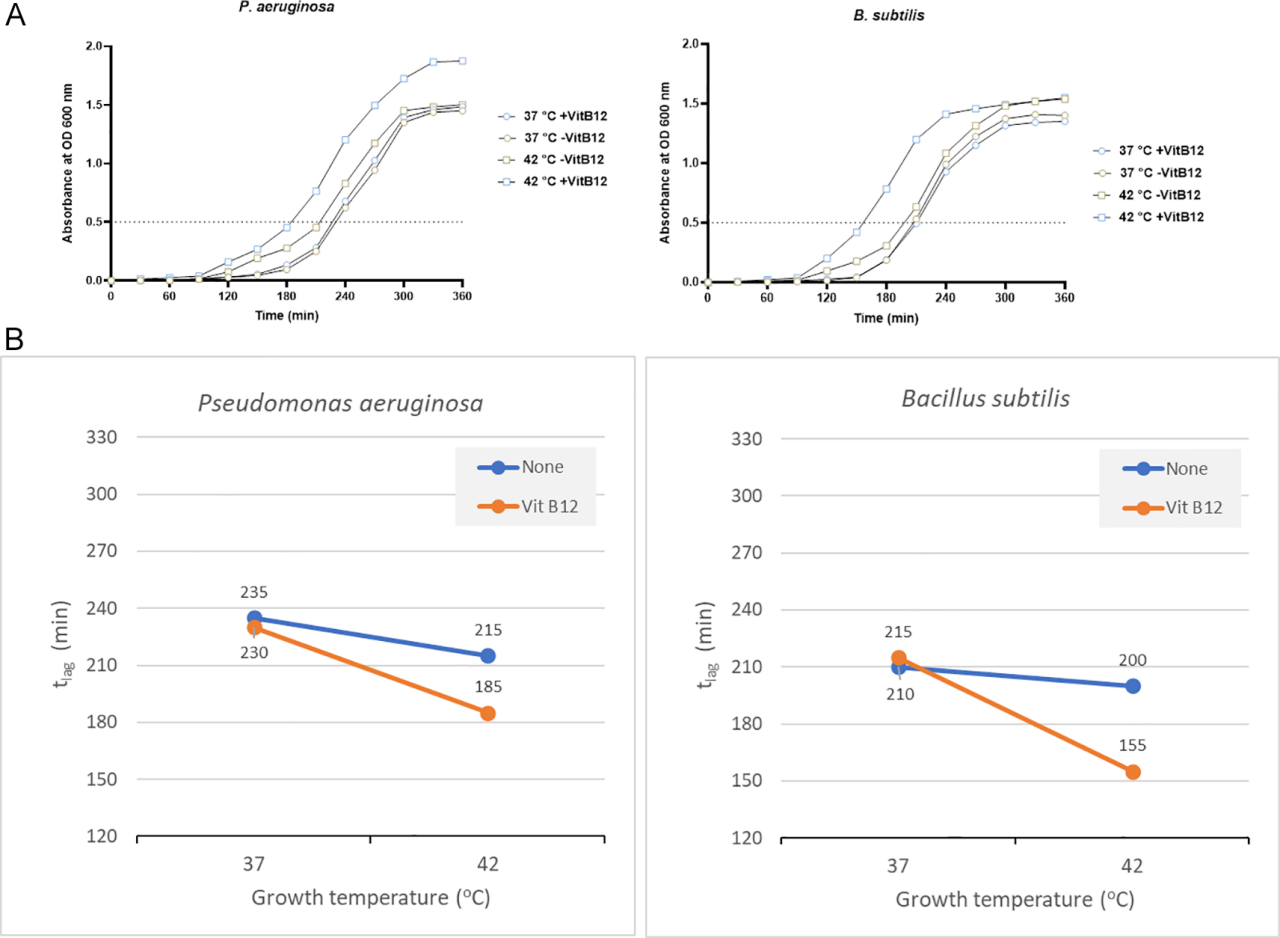
Results for *Pseudomonas aeruginosa* and *Bacillus subtilis* under different growth conditions, including temperature and vitamin B12 supplementation. (A) Growth curves of *P. aeruginosa* and *B. subtilis * were obtained in two-factorial designs, at 37°C and 42°C, with and without vitamin B12. The dotted line indicates OD 0.5 for estimating the t _lag_ from curves. (B) Effect plot of *P. aeruginosa* and *B. subtilis* in minutes (t _lag_) to reach optical density 0.5.

*P. aeruginosa* showed a significant reduction in the t _lag_ when vitamin B12 supplements were added along with 42°C incubation. It reached OD 0.5 in 185 minutes when vitamin B12 supplement was added along with 42°C incubation. On the other hand, 235 minutes was required with no vitamin B12 supplement and 37°C incubation ( Figure 5A).

The effect plot showed a reduction of 30 minutes in t _lag_ when bacteria were cultured at 42°C in the presence of vitamin B12 supplementation ( Figure 5B). The decline in t _lag_ was between 9% and 20% for the four tested conditions.

*Bacillus subtilis*

*B. subtilis* also showed the same pattern as other bacteria, with a t _lag_ of 155 minutes with vitamin B12 supplement and 42°C incubation. On the other hand, the t _lag_ was 210 minutes and 215 minutes to reach OD 0.5 when incubated at 37°C with and without vitamin B12 supplement, respectively ( Figure 5A and Supplementary Table 3 [ *Extended data*]). The effect plot showed a reduction of 45 minutes in t _lag_ when bacteria were co-cultured at 42°C in the presence of vitamin B12 ( Figure 5B). The reduction in t _lag_ was between 5% and 28% for the four tested conditions.

**Figure 6.**
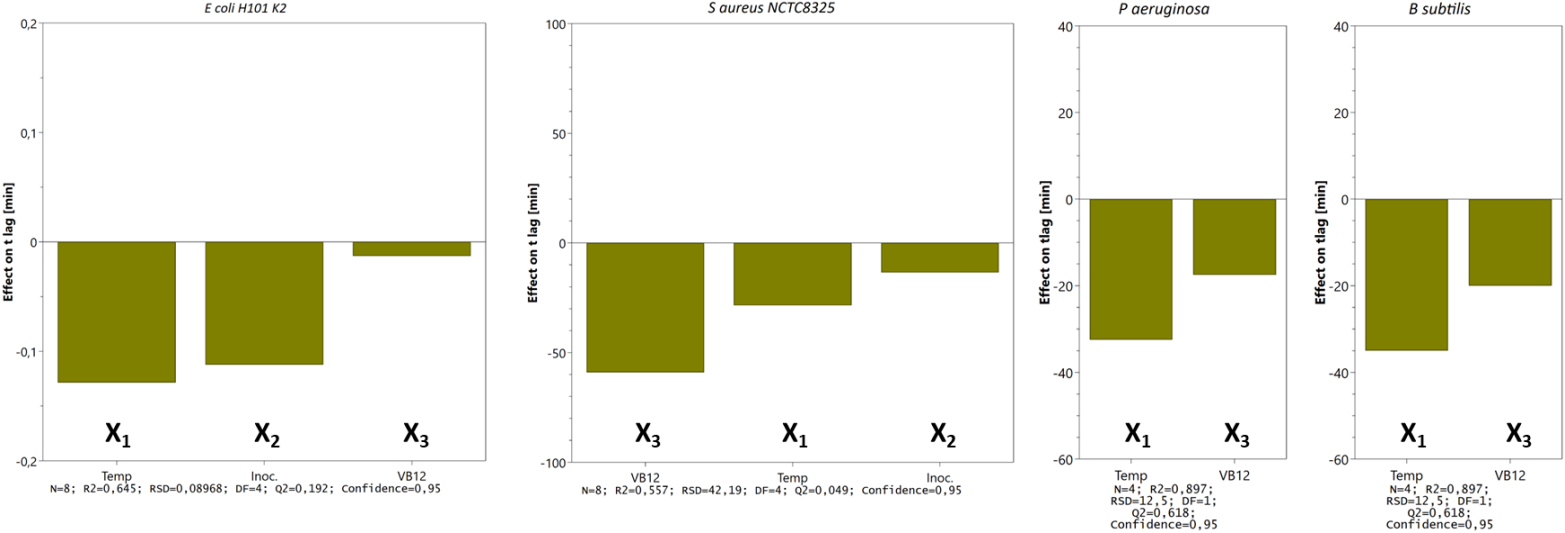
Main effects on t _lag_ from the variables tested in three- ( *E. coli* and *S. aureus*) and two-factor ( *P. aeruginosa* and *B. subtilis*) experimental designs. Bars represent the change in t _lag_ (min) when a factor varies from its low to a high level (cfr. Table 1) when all other factors are kept at their averages. Calculated effects are given with 95% confidence. Other statistical features are given at the bottom.

The main effects on t _lag_ from the variables tested in three- ( *E. coli* and *S. aureus*) and two-factor ( *P. aeruginosa* and *B. subtilis*) experimental designs are shown in Figure 6. We tested the time to reach OD 0.5 for *S. aureus, E. coli, P. aeruginosa,* and *B. subtilis* and noticed that incubating the bacteria at 42°C reduces the lag time. However, the clinical isolates ( *E. coli* CCUG17620, *E. coli* NCTC13441, and *S. aureus* CCUG35600), which were tested for trace metal solution complement, adversely affected bacterial growth by prolonging the lag time (details in *Extended data*).

### Higher incubation temperature increases bacterial growth in blood cultures

**Figure 7.**
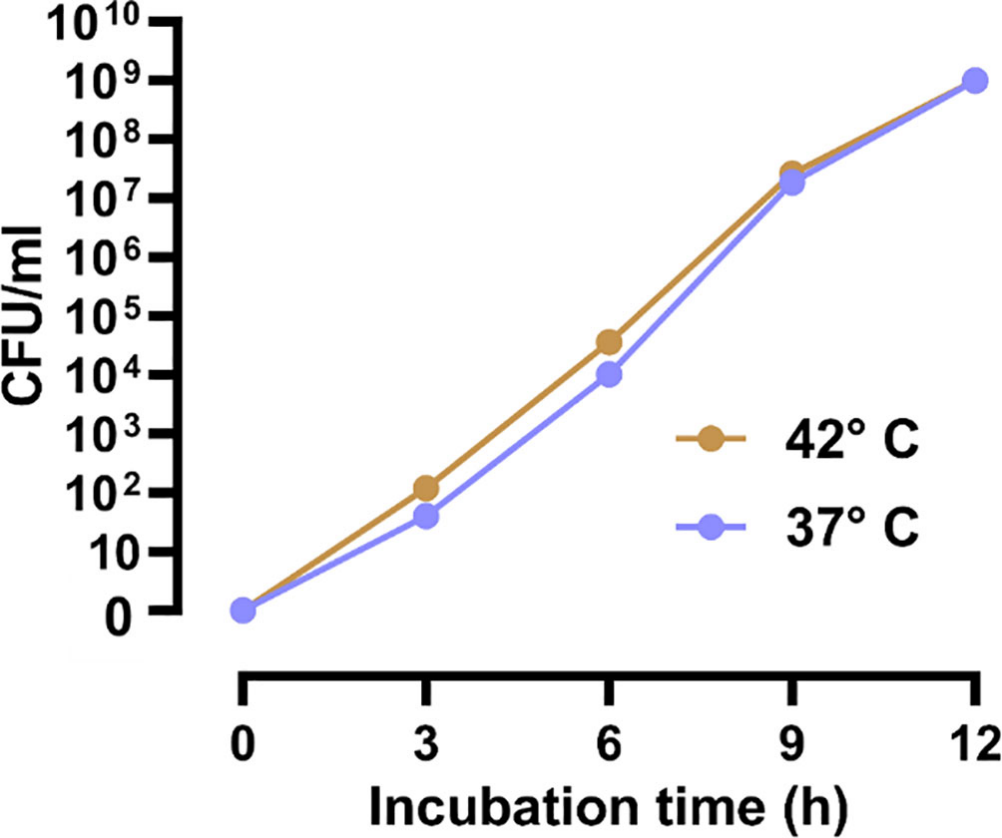
Increased viable counts (CFU/mL) of *E. coli* CCUG17620 spiked in blood cultures cultivated at 37 and 42°C.

We also observed that higher incubation temperature could increase bacterial growth in blood cultures as in pure bacterial isolates. The CFU of the blood samples, which were spiked with *E. coli* CCUG17620 and incubated at the two study temperatures, 37°C and 42°C, showed that bacteria could grow much faster in the early stages of incubation at 42°C compared to 37°C. When we calculated the CFU after 3 h and 6 h of incubation, we could see that the CFU/mL of bacteria were three times higher at 42°C than at 37°C. This higher growth of bacteria in the initial hours of incubation is critical for identifying the causative pathogen. This information is valuable for infection diagnostic purposes. Further incubation beyond 6 h, narrowed the gap between CFU/mL at 37°C and 42°C. At 12 h of incubation, we got the same CFU at both temperatures ( Figure 7). 

### PCR results show higher purity of DNA at an increased growth temperature

**Figure 8.**
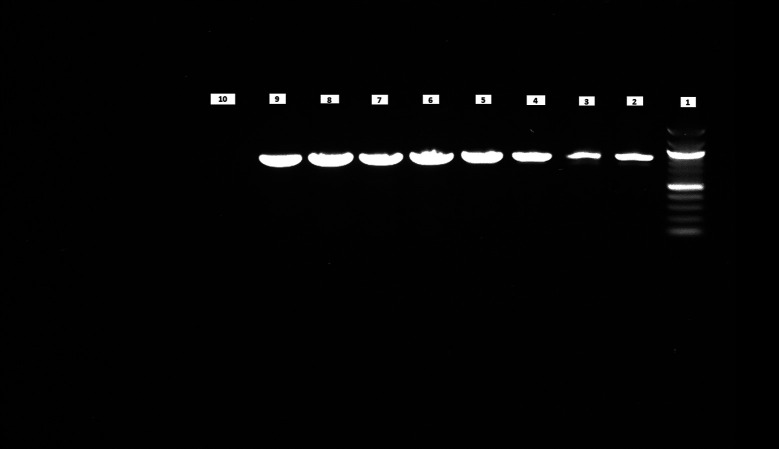
Blood cultures PCR ( *E. coli* CCUG17620). Lane 1: 100 bp DNA ladder, Lane 2: 3 h sample (37°C), Lane 3: 3 h sample (42°C), Lane 4: 6 h sample (37°C), Lane 5: 6 h sample (42°C), Lane 6: 9 h sample (37°C), Lane 7: 9 h sample (42°C), Lane 8: 12 h sample (37°C), Lane 9: 12 h sample (42°C), Lane 10: Negative control (No DNA).

**Figure 9.**
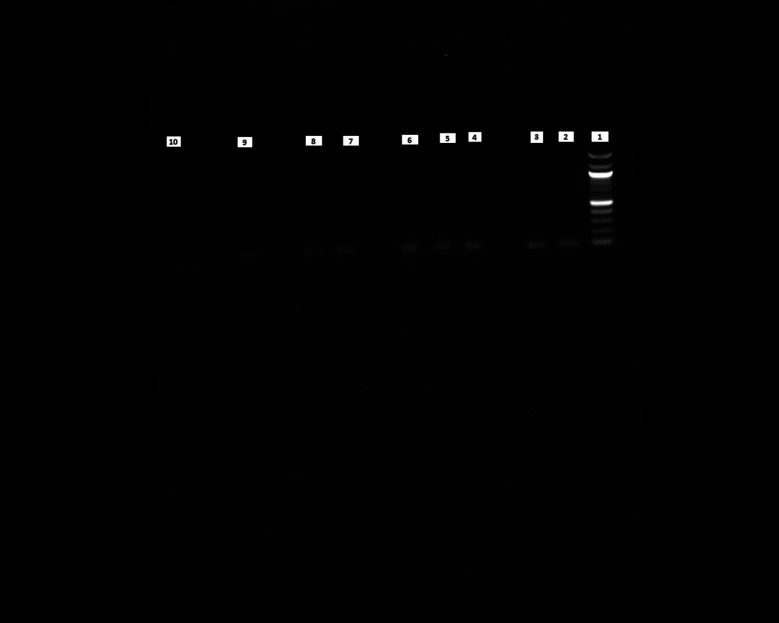
Blood cultures PCR ( *Human*). Lane 1: 100 bp DNA ladder, Lane 2: 3 h sample (37°C), Lane 3: 3 h sample (42°C), Lane 4: 6 h sample (37°C), Lane 5: 6 h sample (42°C), Lane 6: 9 h sample (37°C), Lane 7: 9 h sample (42°C), Lane 8: 12 h sample (37°C), Lane 9: 12 h sample (42°C), Lane 10: Negative control (No DNA).

We performed PCR to confirm the presence of bacterial DNA *versus* human DNA at different incubation time points, as described in Methods. The PCR product run on 1% agarose gel was visualized for the presence of DNA and compared with a 100 bp DNA ladder, which was also run on the gel. We could see the DNA bands for the *E. coli* CCUG 17620 sample even after 3 h incubation. Although the CFU/mL after 3 h of incubation was low, *i.e.*, 40 at 37°C and 120 at 42°C, it was still enough bacterial DNA to be amplified through PCR ( Figure 8). For the samples which were incubated for a longer time and had greater CFU, the band signals for *E. coli* DNA appeared stronger in comparison to the human DNA ( Figures 8 and 9). We can see from the PCR results that we obtained amplification at both the incubation temperatures (37°C and 42°C), but the DNA band strength and purity were higher at 42°C. This higher quality of DNA is beneficial for nanopore sequencing, which can be used to identify the bacteria and ARGs in real time.

## Discussion

Early and appropriate detection of pathogens is crucial for treating bacterial infections. However, since the bacterial load is usually very low for infection detection, it is difficult to identify the pathogen and recommend appropriate antibiotic therapy. In the clinical routine, bacteria are grown overnight, requiring a longer time to grow in fresh culture media. Therefore, any method which increases bacterial growth in the initial hours of culturing can be extremely useful in treating patients with different bacterial infections. In the case of sepsis, early and appropriate antibiotic therapy in the first hour of infection can significantly reduce the mortality rate ( Avershina *et al.*, 2022; Bloos *et al.*, 2017; Ferrer *et al.*, 2014).

The most common method currently used for identifying bacterial pathogens in clinical samples is based on culture. Although these culturing methods, in most cases, provide information on the causative agent, they are slow and time-consuming. The routine culture-based identification takes around 48-72 h, and an additional 24-48 h for antibiotic susceptibility testing. Still, this method is most widely used in clinical diagnosis because it is cost-effective and does not require very advanced lab infrastructure. Many other methods, such as whole genome sequencing (WGS), fluorescent in-situ hybridization (FISH), PCR, and matrix-assisted laser desorption/ionization time-of-flight mass spectrometer (MALDI-TOF MS), has the potential for rapid identification of pathogens. However, most of these techniques still require bacterial culturing as the initial step and currently need to be well-developed to be used in clinical settings.

In this study, we have used different inoculation sizes (0.05% and 0.05%), incubation temperatures (37°C and 42°C), and bacterial growth supplements (vitamin B12 and trace metal solution) to study their effect on the growth of bacteria. We used BHI culture media for bacterial growth because it can support the growth of a wide variety of bacteria. BHI is also suitable for a blood culture medium and is similar to the BACTEC blood culture medium, which is extensively used in the clinical microbiology routine. Also, it has been previously used for short-term incubation of positive blood cultures to accelerate the identification of bacteria by MALDI-TOF MS ( Torres *et al.*, 2017). We have shown that incubating the bacteria at a higher temperature of 42°C instead of 37°C and adding vitamin B12 supplementation in the culture media can significantly reduce the t _lag_ of bacterial growth. We observed the reduced t _lag_ in the pure bacterial isolates and the *E. coli* blood culture using 42°C incubation. The current methods require the blood cultures to be incubated for 24 h or more before identifying pathogens using biochemical tests and then AST. Using this higher incubation temperature (42°C), we can see that after 3 h of incubation, the bacterial CFU/mL was 120, which is three times higher than the standard incubation temperature (37°C). Although the PCR results show bacterial DNA’s presence at both temperatures, we can see the better quality of DNA at 42°C by looking at the intensity of bands ( Figure 8). This high-quality DNA can be beneficial for performing downstream analyses like PCR or real-time metagenomic sequencing for identifying pathogens and ARGs.

The duration of the lag phase is influenced by several factors such as pH, temperature, nutrients, physiological history of the cells, and size of the inoculum ( Bertrand, 2019; Mellefont *et al.*, 2003; Rolfe *et al.*, 2012). Many studies have investigated the effect of environmental factors such as pH, temperature, and nutrients on the duration of the bacterial lag phase ( Francois *et al.*, 2006, 2007). However, so far, no studies have been done to investigate the effect of vitamin B12 supplements on the bacterial lag phase at different conditions, such as temperature (37°C and 42°C) in connection with inoculum size (0.05% and 0.5%). Other studies have also reported that bacterial lag time depends on the nutrient concentration, such as potassium bicarbonate (KHCO _3_), amino acids, ferrous sulfate, sodium pyruvate, and mucin ( Berthelet and MacLeod, 1989). Jiang *et al.* have shown that adding 0.3% mucin to brain heart infusion horse serum (BHI-HS) medium reduced the lag time of *Helicobacter pylori* and enhanced its growth ( Jiang & Doyle, 2000). These results are similar to our study, where we have also shown that vitamin B12 supplement in the BHI medium reduces the lag time of bacteria. Malani *et al.* reported that higher concentrations of sucrose, biotin and pantothenate, lower concentrations of cyanocobalamin ( *i.e.*, vitamin B12), CaSO _4_, and glutathione in a growth medium result in larger bacterial inclusion bodies and product expression ( Malani *et al.*, 2020). The microbial cell is composed mainly of carbon, hydrogen, nitrogen, and oxygen elements. Other elements such as phosphorus, sulfur, calcium, magnesium, potassium, iron, and sodium are equally crucial for the vital activities of bacterial metabolism ( dos Santos *et al.*, 2022). These micronutrients are essential for the enzymatic activities of the bacterial cell and act as prosthetic groups or cofactors for necessary metabolic enzymes ( Hood and Skaar, 2012).

It has also been shown that an extended lag phase is advantageous for bacterial survival and helps in the regrowth of bacteria upon the removal of antibiotics ( Bertrand, 2019). Li *et al.* evaluated the effect of 12 different antibiotics on the lag phase of different environmental bacteria. They observed that tetracycline and quinolone were the most common bacteria that resisted due to the extended lag phase of bacteria ( Li *et al.*, 2017). Therefore, reducing bacterial lag time is even more critical as it can contribute to drug resistance against the clinically relevant antibiotics used to treat bacterial infections. For all four tested bacteria ( *S. aureus, E. coli, P. aeruginosa,* and *B. subtilis* at OD 0.5), we reported that incubating the bacteria at 42°C reduces the lag time (t _lag_).

With an increasing volume of cells in culture media, it takes less time for the bacteria to adapt to the new environment and therefore have a shorter lag time ( Robinson *et al.*, 2001; Aamir *et al.*, 2015). In our study, we observed a short lag time for some of the bacteria by using a larger inoculum size of 0.5%, but in *E. coli* HB101K2 and *S. aureus* NCTC8325, we did not observe the same effect.

In this study, we have shown that using vitamin B12 supplements in culture media and incubation at a higher temperature of 42°C can significantly reduce the t _lag_. This reduction in t _lag_ is significant for rapidly detecting pathogens and early and appropriate antibiotic therapy. We have also shown that incubating *E. coli* blood culture at a higher temperature of 42°C reduces the t _lag_ and enhances the growth of bacteria. It will be beneficial in the future to enrich the culture media with different bacterial growth supplements, which can reduce bacterial lag time and thus allow rapid identification of pathogens. However, to increase the method’s reliability and for any potential future use in clinical microbiology, we need to increase the number of bacterial strains analyzed and focus on additional AMR profiles and clinically relevant pathogens. Moreover, the effect of other elements, such as phosphorus, magnesium, calcium, sodium, and iron, as a bacterial growth supplement will be interesting to explore in future research. These results demonstrate the potential that, unlike other molecular-based detection methods, these measures can be cost-effective and do not need any specialized instruments or expertise.

## Data Availability

Open Science Framework: Ali *et al.*, 2023 F1000 Research, https://doi.org/10.17605/OSF.IO/HYNTQ ( Ahmad, 2023). This project contains the following underlying data:
- Ali et al - Raw Data Blood culture Ecoli CFU.xlsx - Ali et al - Raw data_ growth curve OD and tlag.xlsx - Ali et al Raw_Gel_image_Blood_Culture_Ecoli_primers.tif - Ali et al Raw_Gel_image_Blood_Culture_human_primers.tif Open Science Framework: Ali *et al.* 2023 F1000 Research, https://doi.org/10.17605/OSF.IO/HYNTQ ( Ahmad, 2023). This project contains the following extended data:
- Supplementary_Material.docx Data are available under the terms of the Creative Commons Attribution 4.0 International license (CC-BY 4.0).
