# Supplementary material for: Increased growth temperature and vitamin B12 supplementation reduces the lag time for rapid pathogen identification in BHI agar and blood cultures
Source: F1000Res. 2023 Apr 3;12:131. Originally published 2023 Feb 3. [Version 2] doi: 10.12688/f1000research.129668.2 (PMC10133824; doi:10.12688/f1000research.129668.2)
Supplement: Peer review history

# Peer review: Reviewer response for version 2

**DOI:** 10.5256/f1000research.145967.r168604

**Published:** 2023-04-26

**Authors:** Sarita Mohapatra

**Affiliations:** 1 Department of Microbiology, All India Institute of Medical Sciences, New Delhi, Delhi, India

I have seen the changes in the revised version and now the manuscript is approved from my side.

Is the work clearly and accurately presented and does it cite the current literature?

Yes

If applicable, is the statistical analysis and its interpretation appropriate?

Yes

Are all the source data underlying the results available to ensure full reproducibility?

Yes

Is the study design appropriate and is the work technically sound?

Yes

Are the conclusions drawn adequately supported by the results?

Yes

Are sufficient details of methods and analysis provided to allow replication by others?

Yes

Reviewer Expertise:

Antimicrobial resistance

I confirm that I have read this submission and believe that I have an appropriate level of expertise to confirm that it is of an acceptable scientific standard.

# Peer review: Reviewer response for version 2

**DOI:** 10.5256/f1000research.145967.r168605

**Published:** 2023-04-06

**Authors:** Rashid Nazir

**Affiliations:** 1 Department of Environmental Sciences, Abbottabad Campus, COMSATS University Islamabad, Abbottabad, Khyber Pakhtunkhwa, Pakistan

The authors have satisfactorily revised the manuscript as per the comments raised in version-1.

Is the work clearly and accurately presented and does it cite the current literature?

Yes

If applicable, is the statistical analysis and its interpretation appropriate?

I cannot comment. A qualified statistician is required.

Are all the source data underlying the results available to ensure full reproducibility?

Yes

Is the study design appropriate and is the work technically sound?

Yes

Are the conclusions drawn adequately supported by the results?

Yes

Are sufficient details of methods and analysis provided to allow replication by others?

Yes

Reviewer Expertise:

Microbial interactions, Antimicrobial resistance

I confirm that I have read this submission and believe that I have an appropriate level of expertise to confirm that it is of an acceptable scientific standard.

# Peer review: Reviewer response for version 1

**DOI:** 10.5256/f1000research.142369.r162335

**Published:** 2023-03-06

**Authors:** Rashid Nazir

**Affiliations:** 1 Department of Environmental Sciences, Abbottabad Campus, COMSATS University Islamabad, Abbottabad, Khyber Pakhtunkhwa, Pakistan

The manuscript entitled as “Increased growth temperature and vitamin B12 supplementation reduces the lag time for rapid pathogen identification in BHI agar and blood cultures” published on F1000 Research by Ali *et al.* is an interesting work of applied microbiology. The ms is a proof-of-concept study for future diagnostic improvements. Before its indexing, the following are some comments and observations which are recommended to authors for appropriate response during further revision of their manuscript:

- Please explain the rationale for incubation temperature setting (37 to 42°C) and also provide potential reasons for lag phase changes with/without vitamin B12 in different bacterial stains.
- The bacterial inoculum concentration showed different trends for the lag phase on different temperatures and vitamin B12 supplementation, please discuss different reasons to explain these shifts. Are there any other factors (except the ones studied here) to affect the bacterial lag phase?
- Due to the unique ways in which various bacteria behave and function, the responsiveness and functional capabilities fluctuate as the environment changes. When various environmental factors are activated, not all bacteria or pathogens behave the same way. Some act in opposition to the change, while others react positively. So, how likely is it to expand the observed modification for tens of bacterial pathogens? More precisely, the environmental changes may affect the bacterial physiological functions; in this context, do the tested incubation temperature and vitamin supplementation affect the microbes’ pathogenicity or efficiency as well?
- The nutrients are necessary for the bacteria to grow; some are present in significant amounts, while others are scarce. What is the rationale, for authors, to test B12 out of several microbial nutrients?
- As per the study-observations, increased incubation temperature (42°C) reduced the T _lag_, may it do the same in real life body-temperature situations? Reduced lag time may enhance the pathogens’ survival in compromised immunity scenarios with raised body temperature. Please discuss this practical aspect as well.
- As per the ms title and abstract, the 1 ^st^ impression of the study is more like diagnostic improvements while the introduction start with AMR does not fit in appropriately. 2 ^nd^ paragraph or even the 2 ^nd^ sentence of 2 ^nd^ paragraph might be something to start the introduction and whole AMR scenario may follow in later phase.
- Almost half of the 2 ^nd^ paragraph is on ‘Sepsis’, I think it narrates the examples of different fatal diseases, so please say it so.
- Page-1, 2 - abstract and last paragraph of introduction: Switch the Gram-positive ( *Escherichia coli *and *Pseudomonas aeruginosa*) and Gram-negative ( *Staphylococcus aureus *and *Bacillus subtilis*) bacteria in correct order. Table represented them correctly, though.
- Establish a connecting link of 4 ^th^ paragraph (start with Mesophilic bacteria…) with previous one.
- The 1 ^st^ sentence of the paragraph (starting with ‘The bacterial growth curve…) is sufficient, as the rest is quite basic for the microbiology readers.
- As per the ms, bacterial growth reached the stationary phase (approximately 4-5 h), it would be interesting to see the doubling time of the tested bacteria. I am sure the authors would have calculated.
- Figure 8-9: In English, normal reading/writing is from left to write; please correct it ‘currently from right to left’.

Is the work clearly and accurately presented and does it cite the current literature?

Yes

If applicable, is the statistical analysis and its interpretation appropriate?

I cannot comment. A qualified statistician is required.

Are all the source data underlying the results available to ensure full reproducibility?

Yes

Is the study design appropriate and is the work technically sound?

Yes

Are the conclusions drawn adequately supported by the results?

Yes

Are sufficient details of methods and analysis provided to allow replication by others?

Yes

Reviewer Expertise:

Microbial interactions, Antimicrobial resistance

I confirm that I have read this submission and believe that I have an appropriate level of expertise to confirm that it is of an acceptable scientific standard, however I have significant reservations, as outlined above.

# Response

**Published:** 2023-03-21

**Authors:** Rafi Ahmad

**Affiliations:** Inland Norway University, Norway

We thank the esteemed reviewer for taking the time to provide us with feedback. The reviewer's comments have helped improve the manuscript. We hope that we have addressed the reviewer's comments sufficiently.

- Please explain the rationale for incubation temperature setting (37 to 42°C) and also provide potential reasons for lag phase changes with/without vitamin B12 in different bacterial stains.

**Response: **The incubation temperature (37 & 42°C) was used in this study because these temperatures can easily be maintained in standard laboratory settings and the most commonly used temperature range (37 to 42°C) for the majority of bacterial strains in microbiology. We used the higher (42°C) and lower (37°C) temperatures in this range to compare their effect on the bacterial growth (Gutierrez et al., 2018).

 The relevant text is added in the discussion section of the manuscript (Paragraph 3).   

 The lag time for bacterial growth is dependent on many factors, such as temperature, bacterial strain, and growth medium (availability of nutrients). According to our study, vitamin B12 can affect the lag phase of bacterial growth, possibly because it can contribute to different metabolic activities in bacteria, such as the synthesis of nucleic acid and amino acids. Some bacteria can synthesize vitamin B12, while others require it to be present in the growth medium (LeBlanc et al., 2011). The text has been added in paragraph 4 of the discussion section). If the bacteria do not have vitamin B12 available in the growth medium, they require time to synthesize different enzymes, which is required for the production of vitamin B12 (Sultana et al., 2023). Therefore, the lag time of the bacteria, in that case, is longer.

 The relevant text is added in the discussion section of the manuscript (Paragraph 4).

- The bacterial inoculum concentration showed different trends for the lag phase on different temperatures and vitamin B12 supplementation, please discuss different reasons to explain these shifts. Are there any other factors (except the ones studied here) to affect the bacterial lag phase?

**Response: **The reason we did not see any effect of the inoculum concentration on the lag phase in some of the bacteria may be due to several factors, such as inherent bacterial characteristics, culture growth phase, and adaptation to the environment. Some bacteria may have limited ability to utilize the available nutrients even when the inoculum size is larger. Therefore, the inoculum concentration in some of these bacteria may not have any significant effect on their lag phase (Bidlas et al., 2008).

 The relevant text is added in the discussion section of the manuscript (Paragraph 6).

 Many other factors besides the ones tested here can affect the bacterial lag phase. Some of them are the availability of different nutrients such as carbon, nitrogen, and phosphorus. The pH of the growth medium can also affect the bacterial growth rate and metabolism. Oxygen availability is also important for the optimal growth of bacteria as some bacteria require oxygen for their growth, and others are anaerobic (Bertrand, 2019; Mellefont et al., 2003; Rolfe et al., 2012).

 The relevant text is added in the discussion section of the manuscript (Paragraph 4).

- Due to the unique ways in which various bacteria behave and function, the responsiveness and functional capabilities fluctuate as the environment changes. When various environmental factors are activated, not all bacteria or pathogens behave the same way. Some act in opposition to the change, while others react positively. So, how likely is it to expand the observed modification for tens of bacterial pathogens? More precisely, the environmental changes may affect the bacterial physiological functions; in this context, do the tested incubation temperature and vitamin supplementation affect the microbes’ pathogenicity or efficiency as well?

**Response: **The effect of higher incubation temperature and vitamin B12 supplementation on tens of bacteria is difficult to predict unless we have specific experimental data for each species. But in this study, we have shown that tested bacterial strains show reduced lag time in the presence of vitamin B12 and higher incubation temperature. This method can potentially be tested for other clinically important bacteria as well. The effect of higher incubation temperature and vitamin B12 supplementation on bacterial pathogenicity and efficiency is difficult to generalize because microbial pathogenicity and efficiency are complex phenomena involving many other factors, such as bacterial virulence factors, host-pathogen interactions, and immune responses. In some cases, higher incubation temperature and/or vitamin B12 can enhance bacterial pathogenicity by increasing its growth rate or virulence factor expression, while in other cases, it may have the opposite or no effect at all (Konkel & Tilly, 2000).

 We can hypothesize that faster growth will lead to the rapid spread of the pathogen and, therefore, a more serious infection. As the reviewer has stated, reduced lag time may enhance the pathogens’ survival in compromised immunity scenarios with raised body temperature, which will be more pathogenic.

 The text has been incorporated into the revised manuscript (Paragraph 5 - Discussion).

  

- The nutrients are necessary for the bacteria to grow; some are present in significant amounts, while others are scarce. What is the rationale, for authors, to test B12 out of several microbial nutrients?

**Response: **Bacteria require several nutrients to grow, such as carbon, nitrogen, phosphorus, sulfur, etc. Out of all these nutrients, we selected vitamin B12 for testing because it is cheap and easily available (Fang et al., 2017). In our study, we have also used trace elements to see their effect on bacterial growth (Supplementary data). Also, in future studies, the effect of different nutrients on bacterial growth will be interesting to explore, as we have discussed in the manuscript.

 The text has been incorporated into the revised manuscript (Concluding paragraph - Discussion).

- As per the study-observations, increased incubation temperature (42°C) reduced the Tlag, may it do the same in real life body-temperature situations? Reduced lag time may enhance the pathogens’ survival in compromised immunity scenarios with raised body temperature. Please discuss this practical aspect as well.

**Response: **An increase in the body temperature is usually called fever which is the body’s response to fight any pathogen or infection. In this study, we have shown that higher incubation temperature can reduce the t _lag,_ which will enhance bacterial growth. However, it is difficult to replicate the identical effect as observed in the human body in the lab. According to some studies, bacteria have evolved to regulate their virulence factors due to changes in temperature when they enter from the external environment to the host. Bacteria recognize the signal provided by the temperature and enhance the production of their virulence factors accordingly within the host (Lam et al., 2014). The growth of bacteria at higher temperatures in the human body can be fatal. We can hypothesize that faster growth will lead to the rapid spread of the pathogen and, therefore, a more serious infection.

 The relevant text is added in the discussion (paragraph 5) to highlight this aspect.

- As per the ms title and abstract, the 1st impression of the study is more like diagnostic improvements while the introduction start with AMR does not fit in appropriately. 2nd paragraph or even the 2nd sentence of 2nd paragraph might be something to start the introduction and whole AMR scenario may follow in later phase

**Response: **The text in the manuscript has been updated as per the reviewer's suggestion

- Almost half of the 2nd paragraph is on ‘Sepsis’, I think it narrates the examples of different fatal diseases, so please say it so.

**Response:** Updated in the revised manuscript.

- Page-1, 2 - abstract and last paragraph of introduction: Switch the Gram-positive ( *Escherichia coli* and *Pseudomonas aeruginosa*) and Gram-negative ( *Staphylococcus aureus* and *Bacillus subtilis*) bacteria in correct order. Table represented them correctly, though.

**Response: **Updated in the revised manuscript.

- Establish a connecting link of 4th paragraph (start with Mesophilic bacteria…) with previous one.

**Response: **Updated in the revised manuscript.

- The 1st sentence of the paragraph (starting with ‘The bacterial growth curve…) is sufficient, as the rest is quite basic for the microbiology readers.

**Response: **Updated in the revised manuscript.

- As per the ms, bacterial growth reached the stationary phase (approximately 4-5 h), it would be interesting to see the doubling time of the tested bacteria. I am sure the authors would have calculated.

**Response:** The doubling time (t_d) for bacteria was calculated based on the steepest rise in the exponential phase of the growth curve. The OD (log) and time interval in the exponential phase were selected at two points, which showed a specific growth rate (µ). The following formula was used to calculate the bacterial doubling time.

 t­_d = 0.693 / µ

 In the formula above, 0.693 is constant, and “µ” is the specific growth rate, which is calculated by using the following equation:

 µ= (Ln(OD _2_)-Ln(OD _1_)) / (t _2_-t _1_)

 where;

 Ln is the natural log

 OD _2_ is the OD at the endpoint of the exponential phase

 OD _1_ is the OD at the starting point of the exponential phase

 t _2_ is the time corresponding to OD _2_

 t _1_ is the time corresponding to OD _1_

 Relevant text has been added to the methodology ( *Pre-culture and growth* *procedures*), ****table 1, and results ( *Bacterial doubling time*).

- Figure 8-9: In English, normal reading/writing is from left to write; please correct it ‘currently from right to left’.

**Response: **Figures are updated in the revised manuscript.

# Peer review: Reviewer response for version 1

**DOI:** 10.5256/f1000research.142369.r162338

**Published:** 2023-03-02

**Authors:** Sarita Mohapatra

**Affiliations:** 1 Department of Microbiology, All India Institute of Medical Sciences, New Delhi, Delhi, India

The manuscript compares the effect of growth temperature and VitB12 supplementation in reduction of lag time in pathogen growth. I do have the some comments which needs to be addressed:

1. The experiment has been conducted in only one isolate of four different genus. The difference of the bacterial count has been noticed in the initial period of incubation however, most of the experiments showing similar growth pattern over a longer incubatory period. Hence, more number of isolates need to be tested.
2. *Pseudomonas* *aeruginosa* is known pathogen and its growth at 42°C is taken as one of the identification characters for *P. aeruginosa*. It would be helpful if other Gram-negative bacilli, such as the ESKAPE group of pathogens, were included in the experiment.
3. The Introduction is too long, it should be reduced highlighting the limitations of conventional blood culture technique.
4. How was the CFU measured after 3hr and 6hr incubation?
5. Was DNA quantified? Based on gel picture it is difficult to comment on quantity

Minor comments

1. Correct the gram-positive and gram-negative distribution of bacteria in abstract and other places of manuscript e.g. gram-negative bacilli: *E. coli *& *P. aeruginosa* and gram-positive cocci: *Staphylococcus aureus*, gram-positive bacilli: *Bacillus subtilis*).
2. Full form of CCUG1762 must be written where the term is used first
3. In Table 1 the Gram-stain interpretation should be correctly made as Gram-negative bacilli, or gram-positive bacilli, or Gram-positive cocci, etc.
4. Degree symbol is missing in many places.

Is the work clearly and accurately presented and does it cite the current literature?

Yes

If applicable, is the statistical analysis and its interpretation appropriate?

Yes

Are all the source data underlying the results available to ensure full reproducibility?

Yes

Is the study design appropriate and is the work technically sound?

Yes

Are the conclusions drawn adequately supported by the results?

Yes

Are sufficient details of methods and analysis provided to allow replication by others?

Yes

Reviewer Expertise:

Antimicrobial resistance

I confirm that I have read this submission and believe that I have an appropriate level of expertise to confirm that it is of an acceptable scientific standard, however I have significant reservations, as outlined above.

# Response

**Published:** 2023-03-21

**Authors:** Rafi Ahmad

**Affiliations:** Inland Norway University, Norway

We thank the esteemed reviewer for taking the time to provide us with feedback. The reviewer's comments have helped improve the manuscript. We hope that we have addressed the reviewer's comments sufficiently.

- The experiment has been conducted in only one isolate of four different genus. The difference of the bacterial count has been noticed in the initial period of incubation however, most of the experiments showing similar growth pattern over a longer incubatory period. Hence, more number of isolates need to be tested.

**Response:** In this study, we tested three *E. coli, *two *S. aureus, *and one isolate of *P. aeruginosa *and *B. subtilis*. We agree that bacterial counts fluctuate more during the initial incubation period. However, this difference in the bacterial count during the initial incubation time can be advantageous for quickly detecting pathogens. In our unpublished work, we have observed that in blood cultures, pathogens with higher bacterial counts during the early stages of incubation allow for faster detection using nanopore sequencing. Sequencing at a timely stage enables the identification of pathogens and their antimicrobial resistance, allowing for swift therapeutic intervention. As this was a proof-of-concept study, we could only test available bacterial strains which we already possess – most of them known pathogens. Future studies should focus on testing more pathogens and using different nutrients to see their effect on bacterial growth.

- *Pseudomonas aeruginosa* is known pathogen and its growth at 42°C is taken as one of the identification characters for *P. aeruginosa*. It would be helpful if other Gram-negative bacilli, such as the ESKAPE group of pathogens, were included in the experiment.

**Response: **In this study, we have included 3 *E. coli *isolates (Gram-negative bacilli), *P. aeruginosa, *and 2 *S. aureus *isolates from the ESKAPE group of pathogens, but we agree with the reviewer's suggestion that other pathogens from this group would be interesting to investigate. As this was a proof-of-concept study, we were not able to include all the bacterial strains and were restricted to those which we have in our lab, and some of them are clinically relevant antimicrobial resistant pathogens. In future studies, the focus should be on testing more pathogens with different conditions to have a broader knowledge of the bacterial growth response to different conditions.

- The Introduction is too long, it should be reduced highlighting the limitations of conventional blood culture technique.

**Response: **The suggested changes have been incorporated into the revised version of the manuscript.

- How was the CFU measured after 3hr and 6hr incubation?

**Response:** The samples were taken out from the incubator after 3 and 6 hours, and a small volume was cultured on BHI agar plates, which were then incubated overnight for the calculation of CFU using the formula: number of colonies × dilution factor/volume of culture plated.

 Text has been written in the last sentence of *Blood culturing* in the methods section.

- Was DNA quantified? Based on gel picture it is difficult to comment on quantity

**Response: **Yes, the DNA was quantified, and the details are incorporated into the revised manuscript (Results section: *PCR results*).

**Minor comments**

- Correct the gram-positive and gram-negative distribution of bacteria in abstract and other places of manuscript e.g. gram-negative bacilli: *E. coli* & *P. aeruginosa* and gram-positive cocci: *Staphylococcus aureus*, gram-positive bacilli: *Bacillus subtilis*).

**Response:** The correction has been made in the abstract and introduction section of the revised manuscript.

- Full form of CCUG1762 must be written where the term is used first

**Response: **The suggested change has been incorporated in the revised manuscript.

- In Table 1 the Gram-stain interpretation should be correctly made as Gram-negative bacilli, or gram-positive bacilli, or Gram-positive cocci, etc.

**Response:** The table has been updated with the suggested changes included.

- Degree symbol is missing in many places.

**Response:** Updated in the revised manuscript.
